# Supplementary figures and images for: Diffusion-weighted magnetic resonance sequence and CA125/CEA ratio can be used as add-on tools to ultrasound for the differentiation of ovarian from non-ovarian pelvic masses
Source: PLoS One. 2023 Mar 16;18(3):e0283212. doi: 10.1371/journal.pone.0283212 (PMC10019666; doi:10.1371/journal.pone.0283212)

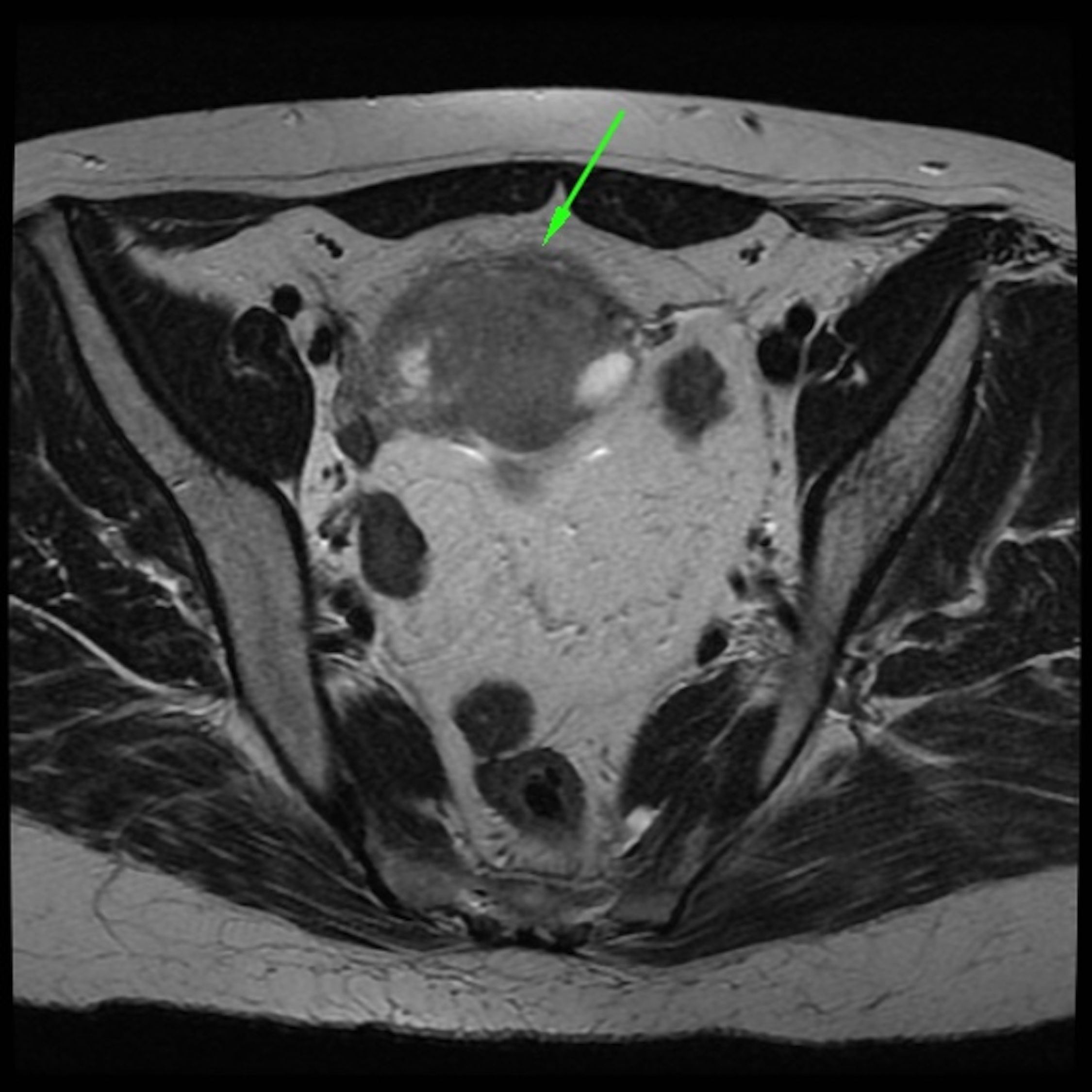

Supplement: S1 Fig — Axial T2 -weighted spin-echo image reveals a right adnexal mass, predominantly solid (green arrow). (TIF) [file pone.0283212.s001.tif]

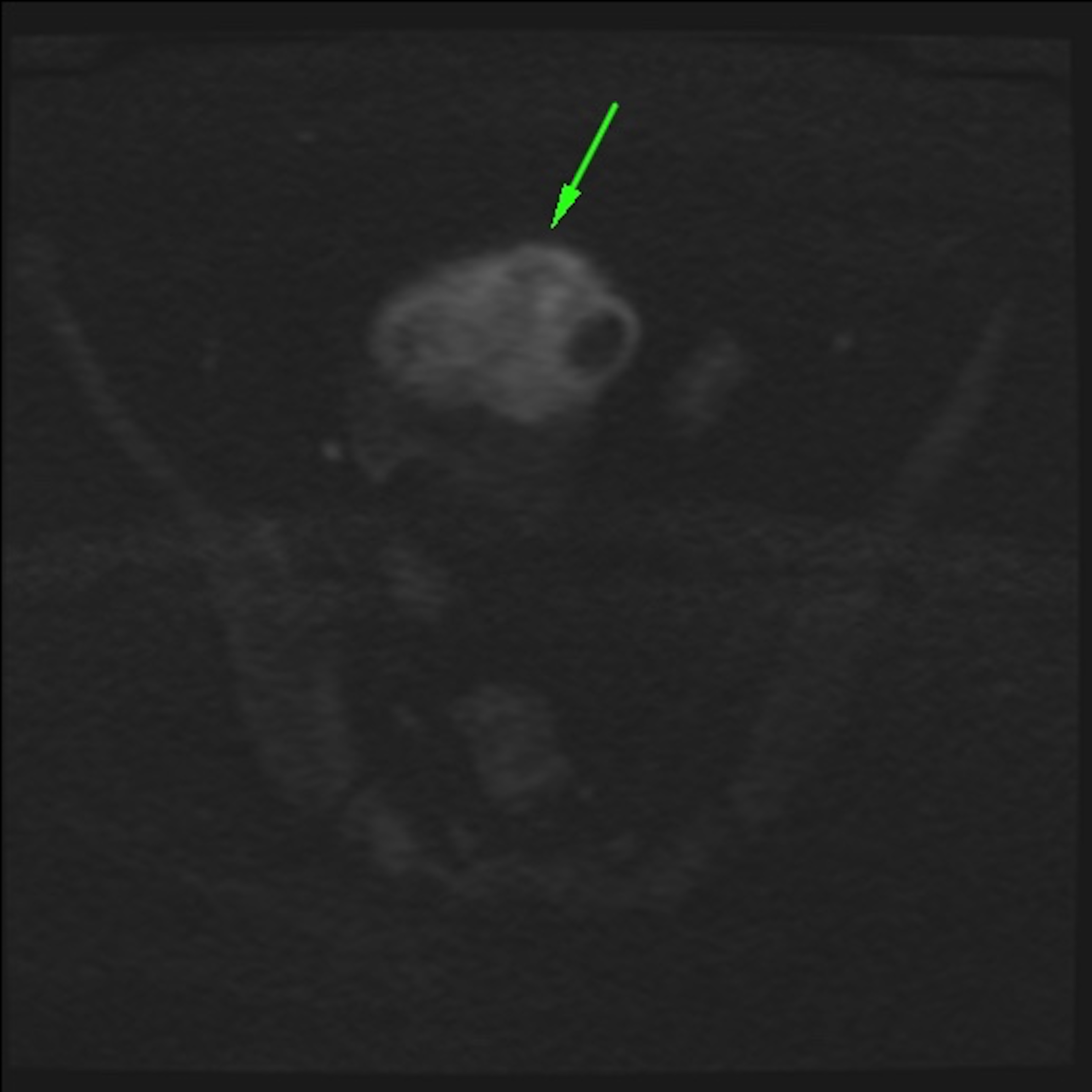

Supplement: S2 Fig — Axial B-1000 diffusion weighted demonstrated areas of high signal in solid tissue in the right adnexal mass (green arrow). CA125/CEA ratio = 75.07 and in the Conditional Inference Tree this tumor was located in group 3 (probability of 100% for ovarian tumor). (TIF) [file pone.0283212.s002.tif]

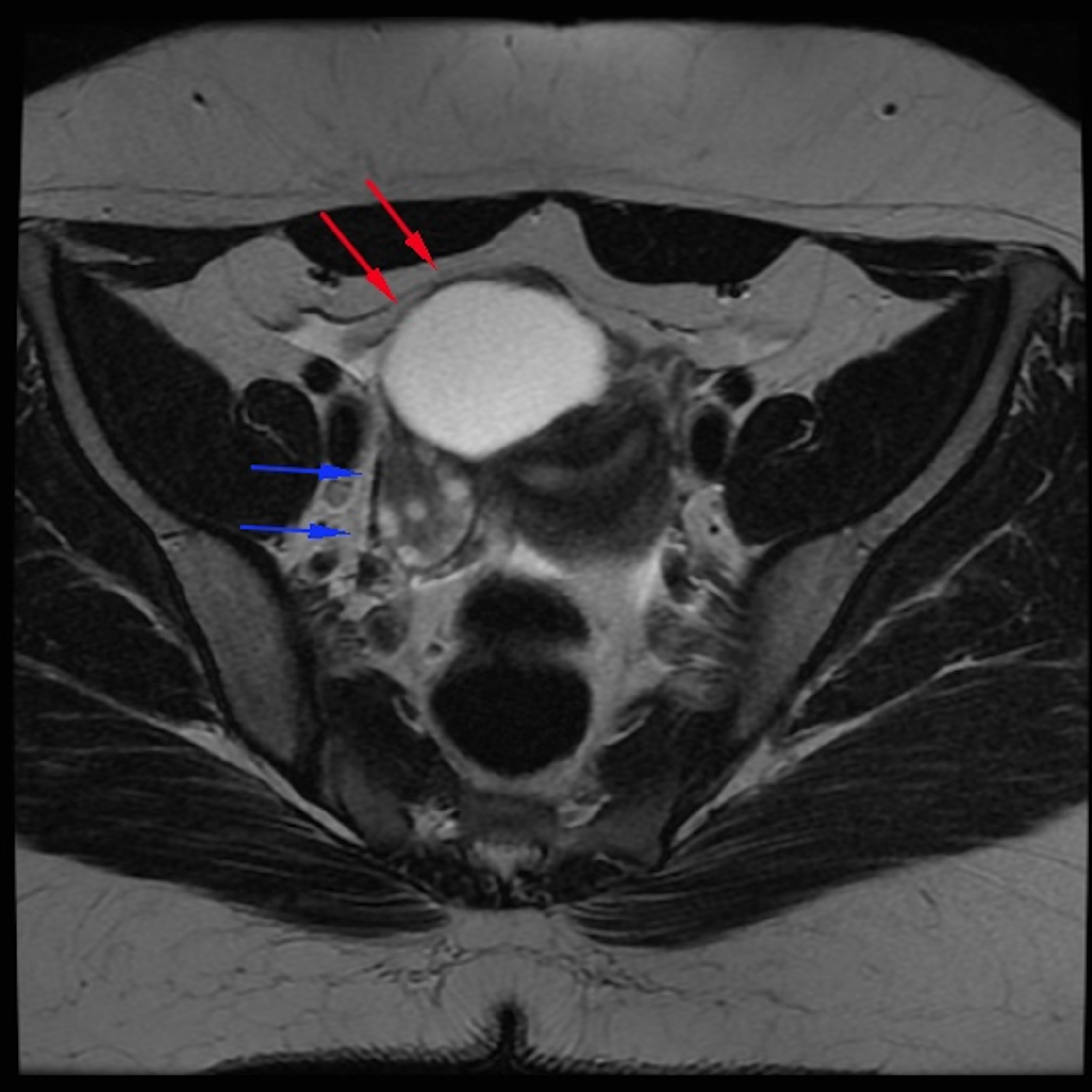

Supplement: S3 Fig — Axial T2 weighted spin-echo image shows an adnexal cystic mass (red arrows), with no septa or solid portion, adhered to the right ovary (blue arrows). It was positioned in group 4 of Conditional Inference Tree. (TIF) [file pone.0283212.s003.tif]

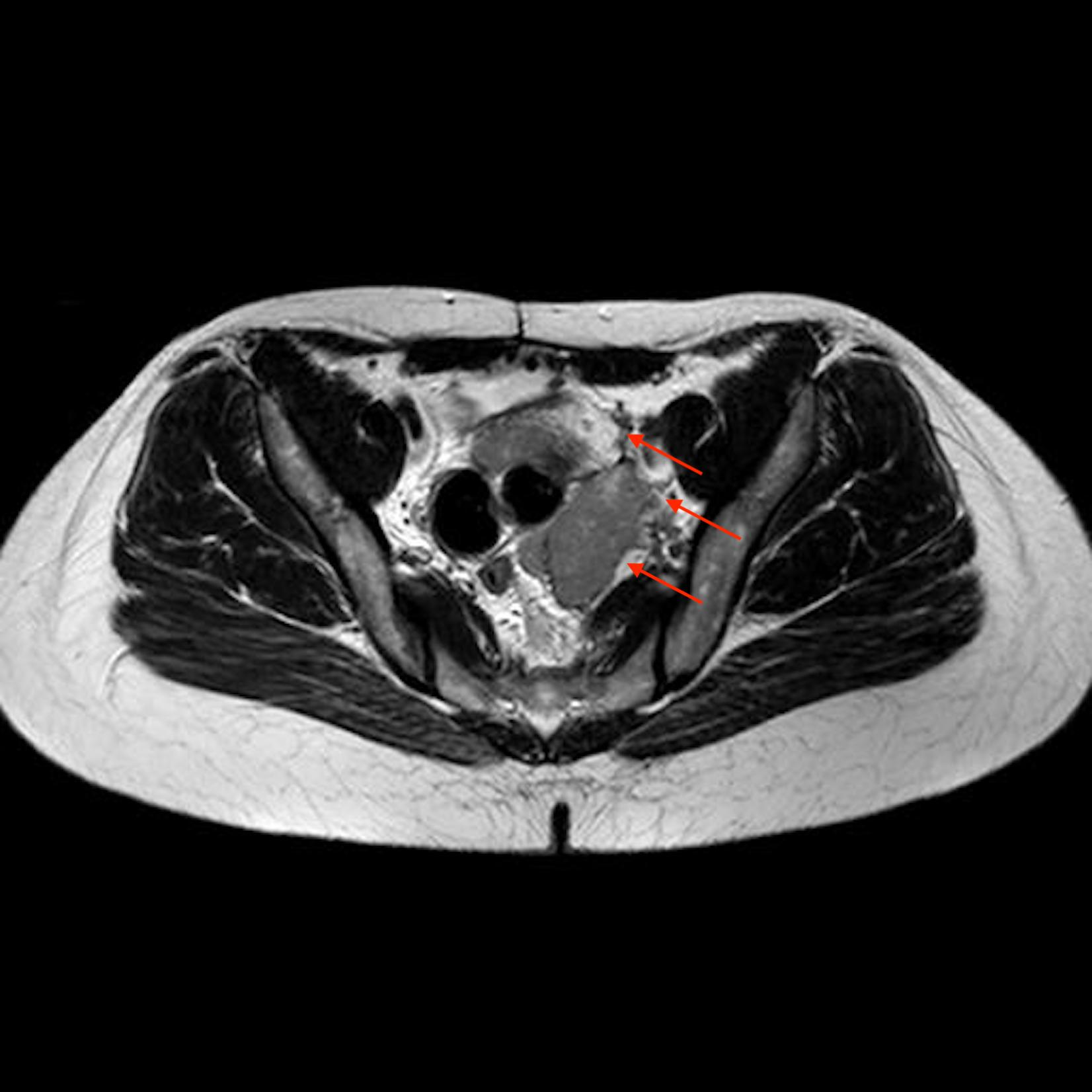

Supplement: S4 Fig — Axial T2 -weighted spin-echo image reveals a left adnexal mass, predominantly solid (red arrows). (TIF) [file pone.0283212.s004.tif]

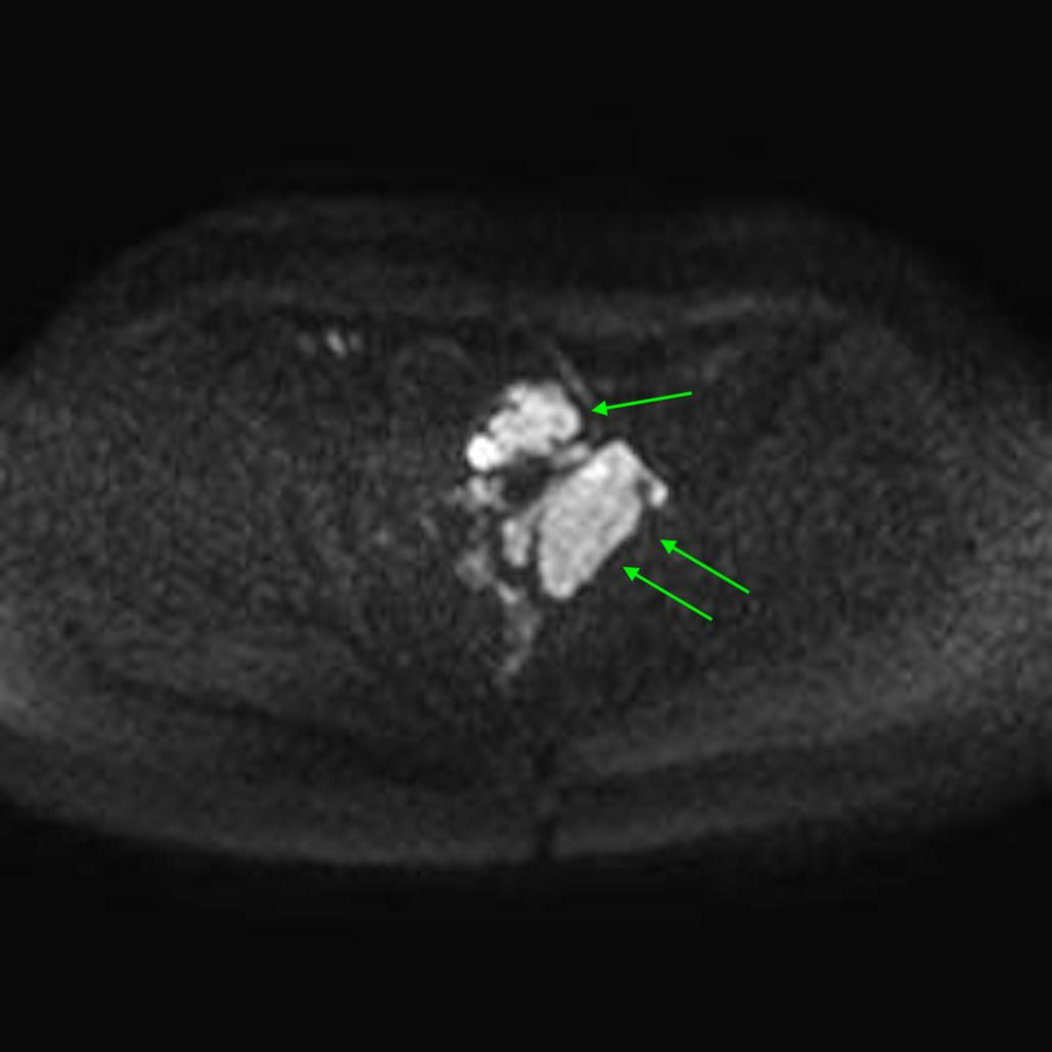

Supplement: S5 Fig — Axial B-1000 diffusion weighted demonstrated multiple areas of high signal in the left adnexal mass (green arrows). Based on conditional inference tree, this mass was categorized in group 2 (probability of 40% for ovarian tumor). (TIF) [file pone.0283212.s005.tif]
